# Graphene-Based Scaffolds: Fundamentals and Applications for Cardiovascular Tissue Engineering

**DOI:** 10.3389/fbioe.2021.797340

**Published:** 2021-12-07

**Authors:** Alex Savchenko, Rose T. Yin, Dmitry Kireev, Igor R. Efimov, Elena Molokanova

**Affiliations:** ^1^ Nanotools Bioscience, La Jolla, CA, United States; ^2^ Department of Biomedical Engineering, The George Washington University, Washington, DC, United States; ^3^ Department of Electrical and Computer Engineering, Microelectronics Research Center, The University of Texas at Austin, Austin, TX, United States; ^4^ Neurano Bioscience, La Jolla, CA, United States

**Keywords:** graphene, scaffold, tissue engineering, biocompability, cardiomyocite, cardiac

## Abstract

Cardiac tissue engineering requires materials that can faithfully recapitulate and support the native *in vivo* microenvironment while providing a seamless bioelectronic interface. Current limitations of cell scaffolds include the lack of electrical conductivity and suboptimal mechanical properties. Here we discuss how the incorporation of graphene into cellular scaffolds, either alone or in combination with other materials, can affect morphology, function, and maturation of cardiac cells. We conclude that graphene-based scaffolds hold great promise for cardiac tissue engineering.

## Introduction

Cardiomyocytes are electrically and mechanically active cells. Therefore, to efficiently support their activities, the cardiac substrates/scaffolds must have matching properties in order to faithfully recapitulate the functional behavior of the myocardium. Despite technological advances, this task remains very challenging, and new materials that can address these problems are in high demand.

**FIGURE 1:**
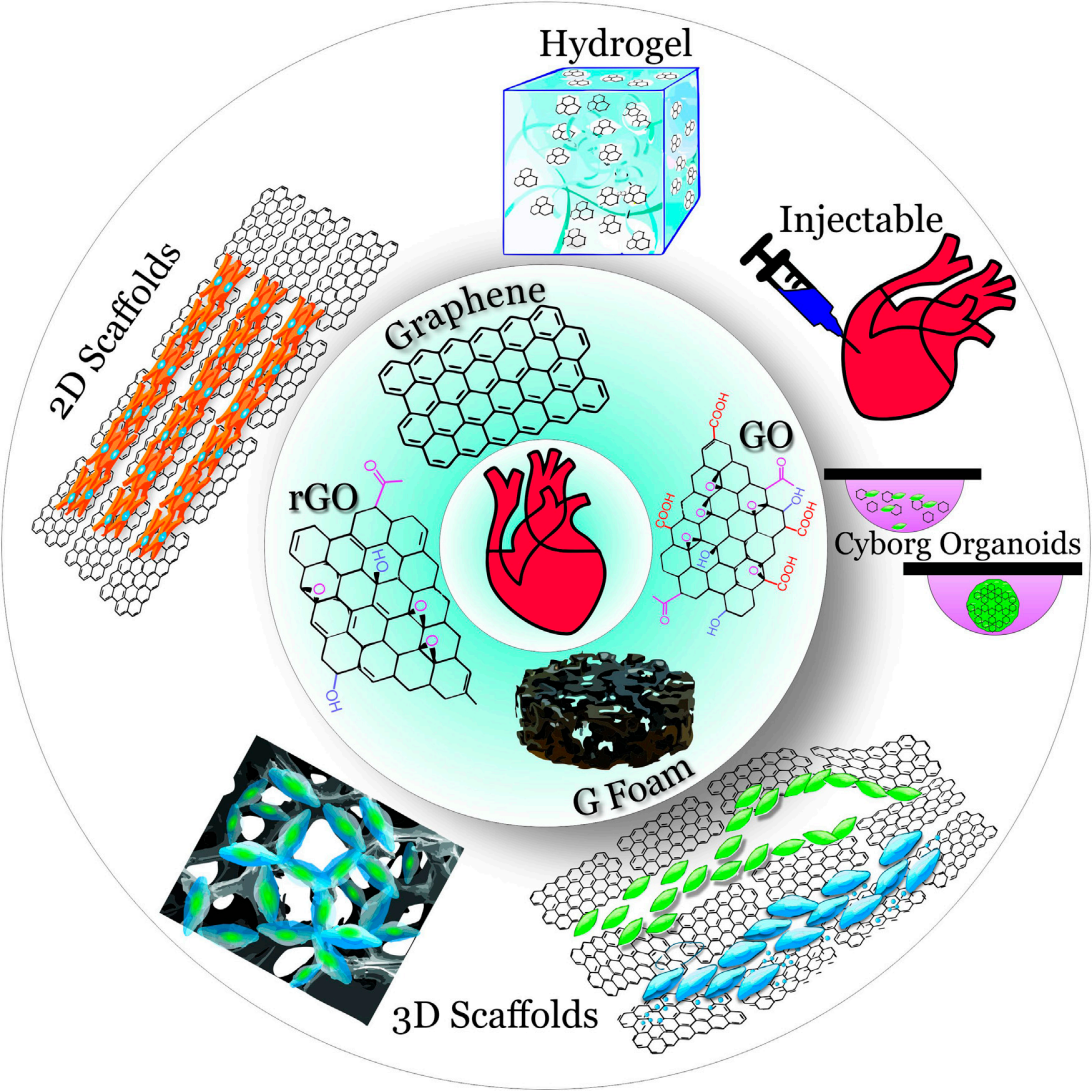
Overview of graphene materials (inner circle) used in 2D and 3D scaffolds (outer circle) for cardiac tissue engineering.

Graphene, the newest member of the carbon allotrope family, is an exceptional candidate for this role. Although other members of this family are already famous [e.g., diamonds (discovered in the 4th century BC in India), graphite (discovered in the 16th century in England), and fullerenes (synthesized in 1985)], graphene could outshine them all. Graphene, a two-dimensional (2D) carbon crystal, is often described using superlatives: graphene is the thinnest (0.335 nm), the lightest (0.77 mg/m2), and the strongest (42 N/m) material (Novoselov et al., 2004; Lee et al., 2008; Geim, 2009; Geim and Novoselov, 2007; Novoselov et al., 2012). Electrical, mechanical, magnetic, optical, and thermal properties of graphene are as just as exceptional, which makes graphene a material unsurpassed in its potential for bioelectronic interfaces and cellular scaffolds. As discussed in this mini-review, the integration of graphene into 2D cell substrates and threedimensional (3D) scaffolds (Figure 1) produces more physiological microenvironment with such features as electrical conductivity, nano-scale topography, stretchability, and flexibility.

## Graphene and its Fundamentals

Graphene came into the spotlight in 2010 with the Nobel Prize in Physics awarded “for groundbreaking experiments regarding the two-dimensional material graphene” (Geim, 2009; Novoselov et al., 2012). It became clear that exceptional physicochemical properties of graphene materials create exciting opportunities for development of novel and efficient graphene-based bioengineering systems.

In graphene, carbon atoms are positioned 0.142 nm apart in a hexagonal lattice. All atoms are sp^2^-hybridized, and each atom allocates 3 electrons from its 4 outer shell electrons to form equivalent covalent σ-bonds with its three neighboring atoms. The 4^th^ electron which occupies a pz orbital is forming a π bond. Unlike the sp2 orbitals, the pz orbitals do not directly overlap, and these 4^th^ electrons (π electrons) operate in the 3^rd^ dimension (above and below a 2D graphene sheet), allowing them to be highly mobile, behave as massless particles, and experience ballistic transport without scattering (Novoselov et al., 2005; Zhang et al., 2005; Geim and Novoselov, 2007; Castro Neto et al., 2009; Tielrooij et al., 2013).

The band structure of pristine graphene consists of a filled valence band and an empty conduction band that cross at the Dirac points. Undoped graphene does not have a band gap and, therefore, is considered a zero-gap semiconductor, or semimetal (Novoselov et al., 2005; Meric et al., 2008). Graphene has very low electrical resistivity, the intrinsic mobility of its electrons is very high (Bolotin et al., 2008) (∼ 200,000 cm^2^/Vs vs. ∼ 1,400 cm^2^/Vs in silicon), and the graphene’s current density is ∼ 1,000,000 times greater than in copper (Chen et al., 2008).

Strong carbon-carbon in-plane σ-bonds (the bonding energy of 4.93 eV) (Brenner and Harrison, 2002) make graphene the strongest material in nature: e.g., the tensile strength of graphene is 130 GPa compared to 0.4 GPa for structural steel or 70 GPa for Kevlar. Composite materials with graphene greatly benefited from its low density (∼2300 kg/m^3^) and large Youngs modulus (∼1 TPa) (Lee et al., 2008). Defect-free graphene sheets exhibit impressive elastic properties and can restore its initial size after strain. Being pliable, graphene can take any form desired. Graphene is also stretchable up to 20% of its initial length.

Graphene oxide (GO) is an oxidized form of graphene containing oxygen functional groups, the presence of which, among other consequences, leads to the disruption of the sp2 structure, increased interlayer spacing, and low electrical conductivity. Reduced graphene oxide (rGO) is a deoxygenized form of GO. Although the graphene structure in rGO is not completely restored by the reduction processes due to some remaining oxygen groups and surface defects, removal of oxygen groups and the increased C/O ratio leads to restoration of the sp2 structure, increased mechanical strength, surface area and stability, hydrophobic properties, and the increase of electric conductivity up to 6300 S cm^−1^ and high mobility of 320 cm^2^ V^−1^ s^−1^ (Smith et al., 2019; Lesiak et al., 2021).

## Biocompatibility

The utmost requirement for any biomedical material is biocompatibility. The very first biological studies of graphene sometimes produced contradictory and/or inconclusive results (Pinto et al., 2013) due to the novelty of graphene materials, the lack of the systematic classification (Wick et al., 2014; Park et al., 2017), and challenges associated with the interdisciplinary nature of nanobiotechnology (Reina et al., 2017). One of the striking examples of biocompatibility controversy surrounding graphene materials is their purported antibacterial properties. Initially, several studies claimed that all graphene materials exhibit antibacterial properties (Akhavan and Ghaderi, 2010; Hu et al., 2010). However, a comprehensive evaluation of graphene samples from different commercial and academic sources by a group from the University of Manchester that included Prof. Novoselov, the Nobel Prize laureate for graphene research, discovered that, in fact, the presence of such cytotoxic impurities as sulfur, boron, and sodium nitrate in graphene samples was the determinant factor for their ability to kill *E. Coli* (Barbolina et al., 2016).

Over subsequent years, it was established that a chemical synthesis route, purity, the number of layers, lateral dimensions, edge effects, defects, doping (intended and unintended), surface functionalization (intended and unintended), and dosage are the factors affecting how graphene may interact with cells (Pang et al., 2017; Seifi and Kamali, 2021). After becoming aware of the criticality of these parameters, scientists can now devise rational bioengineering approaches to synthesize cell-friendly graphene materials and to fabricate biocompatible graphene-based biointerfaces. For example, green-chemistry synthesis methods are highly advisable (Fernández-Merino et al., 2010; Khosroshahi et al., 2018; Regis et al., 2021), and graphene materials should be processed to eliminate various contaminants that might be cytotoxic on their own. Using large-surface graphene-integrated substrates could eliminate such problems as potential physical damage from sharp edges of graphene sheets. Using large (> few microns) graphene flakes or combining of graphene with other materials (e.g., polymers, polysaccharides, hydrogels) can help to avoid endocytosis and potential generation of redox species occurring at the nanoscale.

Currently, the scientific community appears to be in agreement that properly engineered graphene materials are not only biocompatible but often superior in providing a microenvironment necessary for cell growth, differentiation, and development (Kim et al., 2013; Lee et al., 2014; Wang et al., 2017; Kenry et al., 2018).

To evaluate the graphene effects on cell stress, several studies monitored such highly sensitive cell viability indicators as autophagy levels and mitochondrial morphology and membrane potential (Rastogi et al., 2017; Rastogi et al., 2020; Lasocka et al., 2021). These studies determined that graphene has no detectable adverse effect on cell stress and suggested that graphene affects the cell-substrate interactions and promotes cell adhesion and cell proliferation through unique surface topography and adaptable surface chemistry.

Excellent biocompatibility of graphene was also demonstrated in several *in vitro* models of cardiac syncytium (e.g., neonatal rat ventricular cardiomyocytes, mouse embryonic stem cell (ESC)-derived cardiomyocytes, and human induced pluripotent stem cell (hiPSC)-derived cardiomyocytes) cultured on graphene-based planar substrates fabricated from rGO or chemical-vapor-deposition (CVD) graphene. Further, zebrafish embryos injected with graphene flakes (5–20 μm in diameter) were exhibiting the basal heart rates similar to control embryos, and all but one zebrafish retained full viability 3 days later, which confirms biocompatibility of graphene *in vivo* (Savchenko et al., 2018).

## 2D Scaffolds

Graphene substrates are highly biocompatible with monolayer cardiomyocyte cultures (Kim et al., 2013) and appear to be a superior alternative to Matrigel, a substrate commonly used during cardiomyocyte differentiation from stem cells (Hitscherich et al., 2018). Incorporation of graphene into cell substrates was shown to markedly enhance the maturity and electrophysiological properties of hiPSC-cardiomyocytes that otherwise exhibit embryonic rather than adult-like phenotypes. Recent studies evaluating hiPSC-cardiomyocytes cultured on a single graphene layer demonstrated that these cells exhibit a more mature phenotype with improved myofibril alignment and density, increased Cx43 expression, and faster conduction velocity (from 2.2 to 5.3 cm/s). Calcium handling of hiPSC-cardiomyocytes on graphene also became more mature: the expression levels of ryanodine receptors and sarcoendoplasmic reticulum Ca^2+^-ATPase were increased, and calcium transients exhibited a greater amplitude (Wang et al., 2017). This study suggested that a conductive surface of graphene mimicked the heart’s microenvironment and facilitated its intrinsic electrical propagation properties to promote maturation of hiPSC-cardiomyocytes.

Graphene can be combined with other materials to provide multifaceted advantages for hiPSC-cardiomyocytes. One such example is a hybrid collagen/graphene substrate, where collagen provides the biological support while graphene modulates the substrate stiffness and provides the electrical conductivity similar to conductivity of human cardiac tissues. When murine ESC-derived cardiomyocytes were cultured on such substrates (Ryan et al., 2018), they became more aligned and elongated with improved cross-striated sarcomeric structures, suggesting improved maturity.

Hybrid rGO-collagen substrates also produced significant improvements in mechanical and electrical properties of cardiomyocytes in 7 days and led to upregulation of cardiac gene expression involved in electrical coupling (Cx43), muscle contraction and relaxation (troponin-T), and cytoskeleton alignment (actinin-4) even without electrical stimulation (Norahan et al., 2019).

Cardiomyocytes on a vitronectin-coated graphene substrate exhibited enhanced expression of cardiomyogenic markers and cardiac-specific extracellular matrix genes even without addition of cardiomyogenic factors. These results suggest that, by promoting the absorption and correct presentation of vitronectin, graphene induces mesodermal and endodermal lineage signaling which supports the cardiac development (Lee et al., 2014).

Another study transferred graphene films onto polymeric topographic substrates to create a conductive nanopatterned anisotropic scaffold. Cardiomyocytes on this scaffold demonstrated more mature structural and functional properties. These cardiomyocytes exhibited significantly increased z-band widths, greater sarcomere lengths, and increased expression of Cx43. Additionally, the amplitude of calcium transients and the action potential duration were significantly greater, suggesting enhanced maturation of cardiomyocytes (Smith et al., 2017).

## 3D Scaffolds

Since cardiac tissues *in vivo* are inherently 3D, a similar geometric arrangement of engineered heart tissues (EHTs) would be more desirable to ensure faithful recapitulation of cell-to-cell coupling, and complex organization and function. Incorporation of graphene into cardiac 3D scaffolds can lead to anisotropic nanotopology, greatly enhanced electroconductive properties, and provide the ability to perform cell stimulation and monitoring via cell scaffolds.

A graphene foam (Hu et al., 2016; Krueger et al., 2016) itself can serve as a 3D sponge-like scaffold with interconnected pore structure, extended surface area, and nano-micro-scale topographical surface. This scaffold allows for more efficient cell-cell communication and transport of oxygen and nutrients than 2D substrates. Rat neonatal cardiomyocytes on a graphene-enhanced nickel foam were efficiently integrated into the scaffold with adequate biocompatibility and exhibited increased Cx43 gene expression even without direct electrical stimulation (Bahrami et al., 2019). An electrically active graphene foam was also developed to culture cardiomyocytes and directly monitor their extracellular action potentials at the same time (Ameri et al., 2016).

Another option is to directly embed graphene materials into 3D preparations. This approach was shown to increase the number of successfully differentiated 3D embryoid bodies and improve the mechanical and electrical properties of differentiated cardiomyocytes (Ahadian et al., 2016). Furthermore, when electrical stimulation was applied to these graphene-enhanced embryoid bodies, they exhibited more mature sarcomeric structures and became more physiologically active.

Park et al. combined GO flakes (1–6 μm in diameter) with MSCs and discovered that it led to more efficient engrafting of cells at the ischemic lesion site (Park et al., 2015). It appears that the addition of GO may combat the reactive oxygen species abundant in ischemia, prevent anoikis, and improve the therapeutic efficacy of MSCs implantation. This study also determined that paracrine secretion by engrafted cells successfully promoted angiogenesis, alleviated apoptosis in the infarcted region, and decreased infarction size.

Alternatively, cardiomyocytes and graphene materials can be combined to fabricate a complex multilayer cell construct. Shin et al. deposited a poly-L-lysine-GO nanofilm layer onto a homogenous layer of cardiomyocytes, seeded another layer of cells on top of the graphene-enhanced nanofilms, and then repeated these steps until the constructs comprised of 3 or more cell layers (Shin et al., 2014). This scaffold exhibited the elastic modulus of ∼ 10 kPa, resulted in increased expression of *α*-actinin, and supported strong spontaneous synchronous contraction (20–30 BPM).

## Electrospun 3D Scaffolds

Graphene-containing 3D scaffolds can also be fabricated by electrospinning to develop the scaffolds with enhanced mechanical and electrical properties (Zhao et al., 2018; Chen et al., 2019; Ghasemi et al., 2019).

(Hitscherich et al., 2018) employed electrospinning to create a 3D nanofibrous graphene and poly (caprolactone) scaffold where graphene, distributed throughout the scaffold, enabled electrical stimulation of cells. This scaffold enhanced cell-cell coupling and improved the calcium handling in mouse ESC-derived cardiomyocytes as evidenced by increased Cx43 expression, improved cardiomyocyte organization and sarcomere alignment, and significantly increased calcium transient amplitudes and fractional release of calcium ions per beat.

Nazari et al. incorporated rGO-silver nanocomposites into polyurethane nanofibers using used electrospinning and demonstrated that human cardiac progenitor cells cultured on these scaffolds displayed great biocompatibility and cell attachment, and exhibited the upregulation of several cardiac-specific genes (e.g., GATA-4, Tbx18, troponin T, and *α*-MHC) (Nazari et al., 2019).

In another development, poly (caprolactone) (PCL)/poly (glycerol sebacate) (PGS) nanofibers were fabricated via electrospinning, and graphene (0.25, 0.75, or 1% wt) was added to PCL/PGS nanofibers. Addition of graphene to PCL/PGS nanofibrous scaffolds led to improved electrical conductivity, balanced hydrophilicity, and increased surface roughness. Adhesion, growth, migration, proliferation, and viability of cultured human cardiomyocytes were increased with the increase of the ratio of graphene in PCL/PGS scaffolds (Fakhrali et al., 2021).

Talebi et al. used electrospinning technique to fabricate highly hydrophilic fibrous scaffolds composed of polycaprolactone/chitosan/polypyrrole (PCP) and graphene (Talebi et al., 2020). PCP-graphene scaffolds were able to more closely mimic the elasticity and electrical conductivity of the native myocardial tissue, and to support biological and functional performance of murine ESC-derived cardiomyocytes.

To improve nanotopology and electrical conductivity of silk fibroin (SF) scaffolds, rGO nanosheets were incorporated into SF nanofibers *via* electrospinning. When used for cardiac differentiation of human iPSCs transfected with TBX18 gene, rGO-SF scaffolds exhibited improved mechanical and electrical properties, acceptable biocompatibility, considerable cell attachment, enhanced maturity and upregulation of cardiac genes (e.g., GATA-4, c-TnT, and *α*-MHC) (Nazari et al., 2020). Alternatively, a layer of rGO can be deposited on the surface of electrospun SF substrates, also resulting in enhanced electrical conductivity of scaffolds and improved maturity of neonatal rat cardiomyocytes (Zhao et al., 2018).

## Hybrid Hydrogels

The incorporation of graphene materials into hydrogels results in hybrid electroactive scaffolds with improved cell adhesion, desirable mechanical properties, and low immunogenicity.

### *In Vitro* Applications

Zhang et al. used a microcontact printing to create patterned genipin-cross-linked gelatin hydrogels containing GO. When cultured on this substrate, neonatal rat ventricular cardiomyocytes were aligned along the micropatterned scaffolds and demonstrated significant improvements in their structural and functional maturity: 1) more cardiomyocytes were binucleated and had longer sarcomere lengths much sooner; 2) the cardiac gene expression (e.g., *Actn* and *cTnT*) improved; 3) cardiomyocytes reached synchronized contractions within 48 h, contracted in a more uniaxial manner and with increased amplitude, and continued contracting for up to 3 months (Zhang et al., 2019).

rGO can be incorporated into a gelatin methacryloyl (GelMA) hydrogel, which leads to significant enhancement of electrical and mechanical properties of the hybrid material (Shin et al., 2016). Neonatal rat cardiomyocytes on hybrid rGO-GelMA scaffolds exhibited uniformly distributed cell-cell junctions between neighboring cells, better defined and partially uniaxially aligned sarcomeric structures, improved cell viability, proliferation, maturation, stronger contractility, and faster spontaneous contraction rate compared cardiomyocytes to pristine GelMA hydrogels.

Another study utilized GelMA hydrogels with incorporated carbon nanotubes, GO, or rGO to compare the effects of these materials on the structural organization and functionality of hiPSC-cardiomyocytes (Lee et al., 2019). It was determined that electrically conductive rGO-GelMA scaffolds were more efficient in promoting mature morphology of cardiomyocytes, supporting their viability, and increasing the expression of functional cardiac markers than relatively non-conductive GO-GelMA scaffolds. Cardiomyocytes on rGO-GelMA scaffolds exhibited more mature rod-like morphology and higher expression levels of functional cardiac markers (e.g., Cx43 and troponin I), indicative of improved metabolic coupling and more mature excitation-contraction apparatus. Expression of mechanosensors (e.g., integrin, vinculin, and alpha-actinin) was also increased, leading to more robust contractions. Interestingly, cardiomyocytes on GO-GelMA scaffolds exhibited an atrial-like electrophysiological phenotype, while cardiomyocytes on rGO-GelMA scaffolds presented a mixed atrial/ventricular phenotype (Lee et al., 2019).

To engineer advanced EHTs, Tsui et al. used a decellularized extracellular matrix harvested from the left ventricular myocardium of porcine hearts to create a hydrogel composite scaffold with a preserved tissue-specific protein profile and a tunable stiffness enhanced by rGO (Tsui et al., 2021). Mechanical and electrical properties of these hydrogels were tuned by modulating rGO content and degree of reduction. Cardiac tissues engineered with this scaffold showed the increased expression levels of CX43, indicating improved cell-cell connectivity. Multiple functional improvements such as calcium handling, action potential duration, twitch forces, and conduction velocity were also induced by these hybrid rGO-containing scaffolds.

Jing et al. developed polydopamine-based chitosan/GO composite hydrogels and demonstrated that addition of GO increased the hydrogel’s adhesion strength by 300%, and improved its electrical conductivity (Jing et al., 2017). Further, π-π stacking, hydrogen bonding, and supramolecular interactions endowed chitosan/GO hydrogels with high stability, excellent mechanical properties, extended lifespan, self-healing properties, and fast-recovery ability. These hydrogels enhanced cell proliferation and viability and supported faster spontaneous beating rates in hESC-derived cardiomyocytes. These findings were confirmed in another study that tested porous chitosan/GO conductive scaffolds using cardiac H9C2 cell line and established that chitosan/GO scaffolds promoted cell attachment, viability, and upregulation of certain cardiac-specific genes (Jiang et al., 2019).

### *In Vivo* Applications

Myocardial infarction causes irreversible damage to myocardium and results in an increased risk of heart failure and sudden cardiac death. One strategy to address this problem is to integrate new MSCs or cardiomyocytes into the remaining myocardium. Graphene-containing injectable hydrogels could improve the efficiency of engraftment and enhance the survival of implanted cells.

An injectable rGO-enhanced alginate hydrogel was used for culturing MSCs (Karimi Hajishoreh et al., 2020), resulting in the cell viability two-fold higher as compared to a pure alginate or a plastic substrate. Further, rat neonatal cardiac cells encapsulated in the alginate-rGO had significantly upregulated gene expression of *TrpT-2*, *Cx43*, and *Actn4* even without electrical stimulation.

Another study combined methacryloyl-substituted tropoelastin with GO nanoparticles to engineer an injectable, stretchable, conductive hydrogel with an enhanced elasticity and toughness (Annabi et al., 2016). This GO-containing advanced hydrogel exhibited high biocompatibility, promoted the growth and proliferation of cells, and triggered minimal inflammatory response. Neonatal rat cardiomyocytes cultured on this hydrogel developed well-aligned sarcomeric structures that are similar to that of the native ventricular myocardium. Importantly, these cardiomyocytes could be depolarized at a lower excitation threshold, which points to their improved maturity.

To address the loss of vasculature at the ischemic site, Paul et al. employed an injectable graphene-enhanced hydrogel composed of a low-modulus methacrylated gelatin wtih polyethylenimine functionalized GO nanosheets to deliver a proangiogenic vascular endothelial growth factor plasmid DNA to the damaged myocardium (Paul et al., 2014). In a rat model of acute myocardial infarction, this hydrogel significantly decreased the scar area, alleviated inflammation at the infarction site, and improved systolic function as demonstrated by an improved ejection fraction.

Zhao et al. developed an injectable Reverse Thermal Gel (RTG) functionalized with GOs (GO–RTG) and capable of forming a 3D matrix at 37°C (Zhao, 2019). Using neonatal rat ventricular cardiomyocytes, this study demonstrated that these conductive 3D GO–RTG scaffolds can promote cell proliferation and alignment, support long-term survival and maturation, and enhance function properties of cardiomyocytes.

## Conclusion

Numerous reports provided compelling evidence that graphene can play an essential role in creating a more physiologically accurate microenvironment for cardiac cells and EHTs. However, more research needs to be done to optimize the composition and geometry of graphene-based scaffolds for complex long-term *in vivo* applications. It is important to note that the graphene research is still in its infancy, and new fundamental discoveries and new practical applications are emerging every day, further fueling the development of the next wave of biomedical applications. We wholeheartedly share the sentiment expressed by scientists from the University of Manchester, a home of 2010 Nobel Prize Laureates for the research on graphene that “the potential of graphene is limited only by our imagination”.

## References

[B1] Ahadian S. Zhou Y. Yamada S. Estili M. Liang X. Nakajima K. (2016). Graphene Induces Spontaneous Cardiac Differentiation in Embryoid Bodies. Nanoscale 8, 7075–7084. 10.1039/c5nr07059g 26960413

[B2] Akhavan O. Ghaderi E. (2010). Toxicity of Graphene and Graphene Oxide Nanowalls against Bacteria. ACS Nano 4, 5731–5736. 10.1021/nn101390x 20925398

[B3] Ameri S. K. Singh P. K. D'angelo R. Stoppel W. Black L. Sonkusale S. R. (2016). Three Dimensional Graphene Scaffold for Cardiac Tissue Engineering and Iin-Ssitu Electrical Recording. Annu. Int. Conf. IEEE Eng. Med. Biol. Soc. 2016, 4201–4203. 10.1109/EMBC.2016.7591653 28269209

[B4] Annabi N. Shin S. R. Tamayol A. Miscuglio M. Bakooshli M. A. Assmann A. (2016). Highly Elastic and Conductive Human-Based Protein Hybrid Hydrogels. Adv. Mater. 28, 40–49. 10.1002/adma.201503255 26551969 PMC4863466

[B5] Bahrami S. Baheiraei N. Mohseni M. Razavi M. Ghaderi A. Azizi B. (2019). Three-dimensional Graphene Foam as a Conductive Scaffold for Cardiac Tissue Engineering. J. Biomater. Appl. 34, 74–85. 10.1177/0885328219839037 30961432

[B6] Barbolina I. Woods C. R. Lozano N. Kostarelos K. Novoselov K. S. Roberts I. S. (2016). Purity of Graphene Oxide Determines its Antibacterial Activity. 2d Mater. 3, 025025. 10.1088/2053-1583/3/2/025025

[B7] Bolotin K. I. Sikes K. J. Jiang Z. Klima M. Fudenberg G. Hone J. (2008). Ultrahigh Electron Mobility in Suspended Graphene. Solid State. Commun. 146, 351–355. 10.1016/j.ssc.2008.02.024

[B8] Brenner D. W. Shenderova O. A. Harrison J. A. Stuart S. J. Ni B. Sinnott S. B. (2002). A Second-Generation Reactive Empirical Bond Order (REBO) Potential Energy Expression for Hydrocarbons. J. Phys. Condens. Matter 14, 783–802. 10.1088/0953-8984/14/4/312

[B9] Castro Neto A. H. Guinea F. Peres N. M. R. Novoselov K. S. Geim A. K. (2009). The Electronic Properties of Graphene. Rev. Mod. Phys. 81, 109–162. 10.1103/revmodphys.81.109

[B10] Chen J.-H. Jang C. Xiao S. Ishigami M. Fuhrer M. S. (2008). Intrinsic and Extrinsic Performance Limits of Graphene Devices on SiO2. Nat. Nanotech 3, 206–209. 10.1038/nnano.2008.58 18654504

[B11] Chen X. Feng B. Zhu D.-Q. Chen Y.-W. Ji W. Ji T.-J. (2019). Characteristics and Toxicity Assessment of Electrospun Gelatin/PCL Nanofibrous Scaffold Loaded with Graphene *In Vitro* and *In Vivo* . Ijn 14, 3669–3678. 10.2147/ijn.s204971 31190818 PMC6535102

[B12] Fakhrali A. Nasari M. Poursharifi N. Semnani D. Salehi H. Ghane M. (2021). Biocompatible Graphene‐embedded PCL / PGS ‐based Nanofibrous Scaffolds: A Potential Application for Cardiac Tissue Regeneration. J. Appl. Polym. Sci. 138, 51177. 10.1002/app.51177

[B13] Fernández-Merino M. J. Guardia L. Paredes J. I. Villar-Rodil S. Solís-Fernández P. Martínez-Alonso A. (2010). Vitamin C Is an Ideal Substitute for Hydrazine in the Reduction of Graphene Oxide Suspensions. The J. Phys. Chem. C. 114, 6426–6432. 10.1021/jp100603h

[B14] Geim A. K. (2009). Graphene: Status and Prospects. Science 324, 1530–1534. 10.1126/science.1158877 19541989

[B15] Geim A. Novoselov K. (2007). The Rise of Graphene. Nat. Mater. 6, 183–191. 10.1038/nmat1849 17330084

[B17] Ghasemi A. Imani R. Yousefzadeh M. Bonakdar S. Solouk A. Fakhrzadeh H. (2019). Studying the Potential Application of Electrospun Polyethylene Terephthalate/graphene Oxide Nanofibers as Electroconductive Cardiac Patch. Macromol. Mater. Eng. 304, 1900187. 10.1002/mame.201900187

[B18] Hitscherich P. Aphale A. Gordan R. Whitaker R. Singh P. Xie L.-H. (2018). Electroactive Graphene Composite Scaffolds for Cardiac Tissue Engineering. J. Biomed. Mater. Res. 106, 2923–2933. 10.1002/jbm.a.36481 30325093

[B19] Hu C. Xue J. Dong L. Jiang Y. Wang X. Qu L. (2016). Scalable Preparation of Multifunctional Fire-Retardant Ultralight Graphene Foams. ACS Nano 10, 1325–1332. 10.1021/acsnano.5b06710 26745649

[B20] Hu W. Peng C. Luo W. Lv M. Li X. Li D. (2010). Graphene-based Antibacterial Paper. ACS Nano 4, 4317–4323. 10.1021/nn101097v 20593851

[B21] Jiang L. Chen D. Wang Z. Zhang Z. Xia Y. Xue H. (2019). Preparation of an Electrically Conductive Graphene Oxide/chitosan Scaffold for Cardiac Tissue Engineering. Appl. Biochem. Biotechnol. 188, 952–964. 10.1007/s12010-019-02967-6 30740624

[B22] Jing X. Mi H.-Y. Napiwocki B. N. Peng X.-F. Turng L.-S. (2017). Mussel-inspired Electroactive Chitosan/graphene Oxide Composite Hydrogel with Rapid Self-Healing and Recovery Behavior for Tissue Engineering. Carbon 125, 557–570. 10.1016/j.carbon.2017.09.071

[B23] Karimi Hajishoreh N. Baheiraei N. Naderi N. Salehnia M. (2020). Reduced Graphene Oxide Facilitates Biocompatibility of Alginate for Cardiac Repair. J. Bioactive Compatible Polym. 35, 363–377. 10.1177/0883911520933913

[B24] Kenry, Lee W. C. Loh K. P. Lim C. T. (2018). When Stem Cells Meet Graphene: Opportunities and Challenges in Regenerative Medicine. Biomaterials 155, 236–250. 10.1016/j.biomaterials.2017.10.004 29195230

[B25] Khosroshahi Z. Kharaziha M. Karimzadeh F. Allafchian A. (2018). Green Reduction of Graphene Oxide by Ascorbic Acid. AIP Conf. Proc. 1920, 020009. 10.1063/1.5018941

[B26] Kim T. Kahng Y. H. Lee T. Lee K. Kim D. H. (2013). Graphene Films Show Stable Cell Attachment and Biocompatibility with Electrogenic Primary Cardiac Cells. Mol. Cell 36, 577–582. 10.1007/s10059-013-0277-5 PMC3887961 24292978

[B27] Krueger E. Chang A. N. Brown D. Eixenberger J. Brown R. Rastegar S. (2016). Graphene Foam as a Three-Dimensional Platform for Myotube Growth. ACS Biomater. Sci. Eng. 2, 1234–1241. 10.1021/acsbiomaterials.6b00139 28164151 PMC5287265

[B28] Lasocka I. Szulc-Dąbrowska L. Skibniewski M. Skibniewska E. Gregorczyk-Zboroch K. Pasternak I. (2021). Cytocompatibility of Graphene Monolayer and its Impact on Focal Cell Adhesion, Mitochondrial Morphology and Activity in BALB/3T3 Fibroblasts. Materials 14, 643. 10.3390/ma14030643 33573304 PMC7866834

[B29] Lee C. Wei X. Kysar J. W. Hone J. (2008). Measurement of the Elastic Properties and Intrinsic Strength of Monolayer Graphene. Science 321, 385–388. 10.1126/science.1157996 18635798

[B30] Lee J. Manoharan V. Cheung L. Lee S. Cha B.-H. Newman P. (2019). Nanoparticle-based Hybrid Scaffolds for Deciphering the Role of Multimodal Cues in Cardiac Tissue Engineering. ACS nano 13, 12525–12539. 10.1021/acsnano.9b03050 31621284 PMC7068777

[B31] Lee T.-J. Park S. Bhang S. H. Yoon J.-K. Jo I. Jeong G.-J. (2014). Graphene Enhances the Cardiomyogenic Differentiation of Human Embryonic Stem Cells. Biochem. Biophysical Res. Commun. 452, 174–180. 10.1016/j.bbrc.2014.08.062 25152405

[B32] Lesiak B. Trykowski G. Tóth J. Biniak S. Kövér L. Rangam N. (2021). Chemical and Structural Properties of Reduced Graphene Oxide-Dependence on the Reducing Agent. J. Mater. Sci. 56, 3738–3754. 10.1007/s10853-020-05461-1

[B33] Meric I. Han M. Y. Young A. F. Ozyilmaz B. Kim P. Shepard K. L. (2008). Current Saturation in Zero-Bandgap, Top-Gated Graphene Field-Effect Transistors. Nat. Nanotech 3, 654–659. 10.1038/nnano.2008.268 18989330

[B34] Nazari H. Azadi S. Hatamie S. Zomorrod M. S. Ashtari K. Soleimani M. (2019). Fabrication of Graphene‐silver/polyurethane Nanofibrous Scaffolds for Cardiac Tissue Engineering. Polym. Adv. Technol. 30, 2086–2099. 10.1002/pat.4641

[B35] Nazari H. Heirani‐Tabasi A. Hajiabbas M. Khalili M. Shahsavari Alavijeh M. Hatamie S. (2020). Incorporation of Two‐dimensional Nanomaterials into Silk Fibroin Nanofibers for Cardiac Tissue Engineering. Polym. Adv. Technol. 31, 248–259. 10.1002/pat.4765

[B36] Norahan M. H. Pourmokhtari M. Saeb M. R. Bakhshi B. Soufi Zomorrod M. Baheiraei N. (2019). Electroactive Cardiac Patch Containing Reduced Graphene Oxide with Potential Antibacterial Properties. Mater. Sci. Eng. C. 104, 109921. 10.1016/j.msec.2019.109921 31500009

[B37] Novoselov K. S. Fal′ko V. I. Colombo L. Gellert P. R. Schwab M. G. Kim K. (2012). A Roadmap for Graphene. Nature 490, 192–200. 10.1038/nature11458 23060189

[B38] Novoselov K. S. Geim A. K. Morozov S. V. Jiang D. Katsnelson M. I. Grigorieva I. V. (2005). Two-dimensional Gas of Massless Dirac Fermions in Graphene. Nature 438, 197–200. 10.1038/nature04233 16281030

[B39] Novoselov K. S. Geim A. K. Morozov S. V. Jiang D. Zhang Y. Dubonos S. V. (2004). Electric Field Effect in Atomically Thin Carbon Films. Science 306, 666–669. 10.1126/science.1102896 15499015

[B40] Pang L. Dai C. Bi L. Guo Z. Fan J. (2017). Biosafety and Antibacterial Ability of Graphene and Graphene Oxide *In Vitro* and *In Vivo* . Nanoscale Res. Lett. 12, 564. 10.1186/s11671-017-2317-0 29027140 PMC5639822

[B41] Park J. Kim B. Han J. Oh J. Park S. Ryu S. (2015). Graphene Oxide Flakes as a Cellular Adhesive: Prevention of Reactive Oxygen Species Mediated Death of Implanted Cells for Cardiac Repair. ACS nano 9, 4987–4999. 10.1021/nn507149w 25919434

[B42] Park M. V. D. Z. Bleeker E. A. J. Brand W. Cassee F. R. Van Elk M. Gosens I. (2017). Considerations for Safe Innovation: The Case of Graphene. ACS Nano 11, 9574–9593. 10.1021/acsnano.7b04120 28933820

[B43] Paul A. Hasan A. Kindi H. A. Gaharwar A. K. Rao V. T. S. Nikkhah M. (2014). Injectable Graphene Oxide/hydrogel-Based Angiogenic Gene Delivery System for Vasculogenesis and Cardiac Repair. ACS nano 8, 8050–8062. 10.1021/nn5020787 24988275 PMC4148162

[B44] Pinto A. M. Gonçalves I. C. Magalhães F. D. (2013). Graphene-based Materials Biocompatibility: a Review. Colloids Surf. B: Biointerfaces 111, 188–202. 10.1016/j.colsurfb.2013.05.022 23810824

[B45] Rastogi S. K. Bliley J. Matino L. Garg R. Santoro F. Feinberg A. W. (2020). Three-dimensional Fuzzy Graphene Ultra-microelectrodes for Subcellular Electrical Recordings. Nano Res. 13, 1444–1452. 10.1007/s12274-020-2695-y

[B46] Rastogi S. K. Raghavan G. Yang G. Cohen-Karni T. (2017). Effect of Graphene on Nonneuronal and Neuronal Cell Viability and Stress. Nano Lett. 17, 3297–3301. 10.1021/acs.nanolett.7b01215 28383278

[B47] Regis J. Vargas S. Irigoyen A. Bramasco-Rivera E. Bañuelos J. L. Delfin L. C. (2021). Near-UV Light Assisted green Reduction of Graphene Oxide Films through L-Ascorbic Acid. Int. J. Smart Nano Mater. 12, 20–35. 10.1080/19475411.2021.1887396

[B48] Reina G. González-Domínguez J. M. Criado A. Vázquez E. Bianco A. Prato M. (2017). Promises, Facts and Challenges for Graphene in Biomedical Applications. Chem. Soc. Rev. 46, 4400–4416. 10.1039/c7cs00363c 28722038

[B49] Ryan A. J. Kearney C. J. Shen N. Khan U. Kelly A. G. Probst C. (2018). Electroconductive Biohybrid Collagen/pristine Graphene Composite Biomaterials with Enhanced Biological Activity. Adv. Mater. 30, 1706442. 10.1002/adma.201706442 29504165

[B50] Savchenko A. Cherkas V. Liu C. Braun G. B. Kleschevnikov A. Miller Y. I. (2018). Graphene Biointerfaces for Optical Stimulation of Cells. Sci. Adv. 4, eaat0351. 10.1126/sciadv.aat0351 29795786 PMC5959318

[B51] Seifi T. Kamali A. R. (2021). Anti-pathogenic Activity of Graphene Nanomaterials: A Review. Colloids Surf. B: Biointerfaces 199, 111509. 10.1016/j.colsurfb.2020.111509 33340933

[B52] Shin S. R. Aghaei-Ghareh-Bolagh B. Gao X. Nikkhah M. Jung S. M. Dolatshahi-Pirouz A. (2014). Layer-by-Layer Assembly of 3D Tissue Constructs with Functionalized Graphene. Adv. Funct. Mater. 24, 6136–6144. 10.1002/adfm.201401300 25419209 PMC4235968

[B53] Shin S. R. Zihlmann C. Akbari M. Assawes P. Cheung L. Zhang K. (2016). Reduced Graphene Oxide‐GelMA Hybrid Hydrogels as Scaffolds for Cardiac Tissue Engineering. Small 12, 3677–3689. 10.1002/smll.201600178 27254107 PMC5201005

[B54] Smith A. S. T. Yoo H. Yi H. Ahn E. H. Lee J. H. Shao G. (2017). Micro- and Nano-Patterned Conductive Graphene-PEG Hybrid Scaffolds for Cardiac Tissue Engineering. Chem. Commun. 53, 7412–7415. 10.1039/c7cc01988b PMC5548490 28634611

[B55] Smith A. T. Lachance A. M. Zeng S. Liu B. Sun L. (2019). Synthesis, Properties, and Applications of Graphene Oxide/reduced Graphene Oxide and Their Nanocomposites. Nano Mater. Sci. 1, 31–47. 10.1016/j.nanoms.2019.02.004

[B56] Talebi A. Labbaf S. Karimzadeh F. Masaeli E. Nasr Esfahani M.-H. (2020). Electroconductive Graphene-Containing Polymeric Patch: a Promising Platform for Future Cardiac Repair. ACS Biomater. Sci. Eng. 6, 4214–4224. 10.1021/acsbiomaterials.0c00266 33463338

[B57] Tielrooij K. J. Song J. C. W. Jensen S. A. Centeno A. Pesquera A. Zurutuza Elorza A. (2013). Photoexcitation cascade and Multiple Hot-Carrier Generation in Graphene. Nat. Phys 9, 248–252. 10.1038/nphys2564

[B58] Tsui J. H. Leonard A. Camp N. D. Long J. T. Nawas Z. Y. Chavanachat R. (2021). Tunable Electroconductive Decellularized Extracellular Matrix Hydrogels for Engineering Human Cardiac Microphysiological Systems. Biomaterials 272, 120764. 10.1016/j.biomaterials.2021.120764 33798964 PMC8074529

[B59] Wang J. Cui C. Nan H. Yu Y. Xiao Y. Poon E. (2017). Graphene Sheet-Induced Global Maturation of Cardiomyocytes Derived from Human Induced Pluripotent Stem Cells. ACS Appl. Mater. Inter. 9, 25929–25940. 10.1021/acsami.7b08777 28718622

[B60] Wick P. Louw-Gaume A. E. Kucki M. Krug H. F. Kostarelos K. Fadeel B. (2014). Classification Framework for Graphene-Based Materials. Angew. Chem. Int. Ed. 53, 7714–7718. 10.1002/anie.201403335 24917379

[B61] Zhang F. Zhang N. Meng H.-X. Liu H.-X. Lu Y.-Q. Liu C.-M. (2019). Easy Applied Gelatin-Based Hydrogel System for Long-Term Functional Cardiomyocyte Culture and Myocardium Formation. ACS Biomater. Sci. Eng. 5, 3022–3031. 10.1021/acsbiomaterials.9b00515 33405656

[B62] Zhang Y. Tan Y.-W. Stormer H. L. Kim P. (2005). Experimental Observation of the Quantum Hall Effect and Berry's Phase in Graphene. Nature 438, 201–204. 10.1038/nature04235 16281031

[B63] Zhao G. Qing H. Huang G. Genin G. M. Lu T. J. Luo Z. (2018). Reduced Graphene Oxide Functionalized Nanofibrous Silk Fibroin Matrices for Engineering Excitable Tissues. NPG Asia Mater. 10, 982–994. 10.1038/s41427-018-0092-8

[B64] Zhao L. (2019). A Novel Graphene Oxide Polymer Gel Platform for Cardiac Tissue Engineering Application. 3 Biotech. 9, 401–411. 10.1007/s13205-019-1912-4 PMC6800416 31681522

